# Nivolumab With or Without Ipilimumab in Patients With Recurrent or Metastatic Merkel Cell Carcinoma: A Nonrandomized, Open-Label, International, Multicenter Phase I/II Study

**DOI:** 10.1200/JCO-24-02138

**Published:** 2025-01-31

**Authors:** Shailender Bhatia, Suzanne L. Topalian, William Sharfman, Tim Meyer, Neil Steven, Christopher D. Lao, Lorena Fariñas-Madrid, Lot A. Devriese, Kathleen Moore, Robert L. Ferris, Yoshitaka Honma, Ileana Elias, Anjaiah Srirangam, Charlie Garnett-Benson, Michelle Lee, Paul Nghiem

**Affiliations:** 1 Division of Hematology-Oncology, University of Washington and Fred Hutchinson Cancer Center, Seattle, WA; 2 Johns Hopkins Bloomberg-Kimmel Institute for Cancer Immunotherapy and Sidney Kimmel Comprehensive Cancer Center, Baltimore, MD; 3 Department of Oncology, University College London Cancer Institute, London, United Kingdom; 4 Institute of Immunology and Immunotherapy, College of Medical and Dental Sciences, University of Birmingham, Birmingham, United Kingdom; 5 Michigan Medicine, Rogel Cancer Center, Ann Arbor, MI; 6 Vall d’Hebron University Hospital, Vall d’Hebron Institute of Oncology, Barcelona, Spain; 7 Department of Medical Oncology, Cancer Center, University Medical Center Utrecht, Utrecht, the Netherlands; 8 Department of Obstetrics and Gynecology, Stephenson Cancer Center at the University of Oklahoma HSC, Oklahoma City, OK; 9 Lineberger Comprehensive Cancer Center, University of North Carolina, Chapel Hill, NC; 10 Department of Head and Neck, Esophageal Medical Oncology, National Cancer Center Hospital, Tokyo, Japan; 11 Bristol Myers Squibb, Princeton, NJ; 12 Syneos Health, Morrisville, NC; 13 University of Washington Medical Center, Seattle, WA

## Abstract

**PURPOSE:**

Approximately 50% of patients with advanced Merkel cell carcinoma (MCC) have primary or acquired resistance to PD-(L)1 blockade, which may be overcome using combination immune checkpoint inhibition (ICI) with anti–cytotoxic T lymphocyte antigen-4 antibody. We present results from the recurrent/metastatic MCC cohort in CheckMate 358, a nonrandomized, multicohort, phase I/II study of nivolumab (NIVO) with or without ipilimumab (IPI) in virus-associated cancers (ClinicalTrials.gov identifier: NCT02488759).

**METHODS:**

ICI-naïve patients with recurrent/metastatic MCC and 0–2 previous systemic therapies were administered NIVO monotherapy at 240 mg once every 2 weeks or combination therapy with NIVO 3 mg/kg once every 2 weeks + IPI 1 mg/kg once every 6 weeks. The primary end point was objective response. Secondary end points included duration of response (DOR), progression-free survival (PFS), and overall survival (OS).

**RESULTS:**

Sixty-eight patients received NIVO (n = 25) or NIVO + IPI (n = 43). The objective response rate (95% CI) and median DOR (95% CI), respectively, were 60% (38.7 to 78.9) and 60.6 months (16.7 to not applicable [NA]) with NIVO and 58% (42.1 to 73) and 25.9 months (10.4 to NA) with NIVO + IPI. The median PFS (95% CI) and OS (95% CI), respectively, were 21.3 (9.2 to 62.5) and 80.7 (23.3 to NA) months with NIVO and 8.4 (3.7 to 24.3) and 29.8 (8.5 to 48.3) months with NIVO + IPI. The incidence of grade 3/4 treatment-related adverse events was 28% with NIVO and 47% with the combination.

**CONCLUSION:**

This nonrandomized study showed frequent and durable responses with both NIVO and NIVO + IPI in patients with ICI-naïve advanced MCC. However, it did not show improvement in efficacy with the combination, thus contradicting previous study reports that had suggested clinical benefit with combination ICI. A randomized trial of NIVO + IPI versus NIVO monotherapy is warranted.

## INTRODUCTION

Merkel cell carcinoma (MCC) is a rare and aggressive form of skin cancer associated with the Merkel cell polyomavirus and ultraviolet radiation exposure.^1^ MCC is regarded as a highly immunogenic cancer^2^ because of the expression of viral antigens and neoantigens in cancer cells.^1,3^ However, despite this immunogenicity, MCC cells can evade the immune system through multiple mechanisms, including the expression of immune checkpoint molecules. The PD-1 immune checkpoint pathway plays an important role in immune evasion by MCC cells, with high expression of PD-L1 in the MCC microenvironment, on cancer cells and/or tumor-infiltrating immune cells.^3^

Several drugs that block the PD-(L)1 pathway, including avelumab, pembrolizumab, and retifanlimab, are US Food and Drug Administration (FDA)–approved for patients with advanced MCC and have been associated with the marked improvement in clinical outcomes as compared with historical outcomes with chemotherapy.^4–9^ An objective response rate (ORR) of 46%-58% was reported in phase II studies of pembrolizumab or retifanlimab.^8,10,11^ However, approximately 50% of patients receiving anti–PD-(L)1 as a first-line (1L) systemic treatment show either primary or acquired resistance, suggesting a need for more effective systemic treatments.^8,10–13^

Ipilimumab (IPI), an anti–cytotoxic T lymphocyte antigen-4 (CTLA-4) antibody, alone or in combination with PD-(L)1 inhibitors, has been reported to be effective in patients with advanced MCC refractory to previous anti–PD-(L)1 therapy, with predominantly small retrospective case series reporting ORRs of 0%-50%.^14–16^ The only published prospective trial of IPI in patients with metastatic MCC investigated the combination of nivolumab (NIVO) (240 mg once every 2 weeks) + IPI (1 mg/kg once every 6 weeks), with or without stereotactic body radiation therapy, and reported an ORR of 100% among 24 immune checkpoint inhibition (ICI)–naïve patients and 31% among 26 patients previously treated with anti–PD-(L)1 therapy.^17^

Here, we present results from the recurrent/metastatic MCC cohorts enrolled in the phase II part of CheckMate 358, a multicenter, international, nonrandomized phase I/II study of NIVO with or without IPI in virus-associated cancers (ClinicalTrials.gov identifier: NCT02488759).

## METHODS

### Study Design and Participants

CheckMate 358 is a multicenter, open-label, multicohort phase I/II trial that investigated NIVO-based therapies in patients with virus-associated solid tumors, such as MCC, in the neoadjuvant or recurrent/metastatic setting. Patients in the recurrent/metastatic MCC cohorts received NIVO monotherapy at 240 mg once every 2 weeks or combination therapy with NIVO 3 mg/kg once every 2 weeks + IPI 1 mg/kg once every 6 weeks. These two cohorts were enrolled sequentially, with the NIVO monotherapy cohort closing to enrollment before opening the NIVO + IPI cohort. Treatment could continue after disease progression as long as the following five conditions were met: investigator-assessed clinical benefit without rapid disease progression, tolerance of study drug as defined by the investigator, stable Eastern Cooperative Oncology Group performance status (ECOG PS), treatment beyond progression would not delay an imminent intervention to prevent serious complications of disease progression (eg, CNS metastases), and the patient provided a new written informed consent. Adverse events requiring treatment delay or discontinuation are listed in the Protocol (online only). Eligible patients had histologically confirmed recurrent/metastatic MCC, had received 0–2 previous systemic therapies for recurrent/metastatic disease, had no previous exposure to immune system–modulating drugs (eg, experimental antitumor vaccines, any T-cell costimulation or checkpoint pathway agents [including anti-PD-(L)1, anti-PD-L2, anti-CD137, or anti-CTLA-4], or other medications specifically targeting T cells), and had an ECOG PS of 0–1. Patients were ineligible if they had active brain metastases or leptomeningeal metastases, another invasive malignancy within 3 years of study enrollment, a history of autoimmune disease, previous treatment with T-cell–modulating drugs, or a requirement for systemic immunosuppressive medications.

### Study End Points

The primary end point for the recurrent/metastatic MCC cohort of the CheckMate 358 study was objective response, as assessed by the investigators per RECIST v1.1. ORR was defined as the proportion of treated patients with a best overall response (BOR) of confirmed complete response (CR) or partial response (PR). BOR was defined as the best response observed between the date of first study drug administration and the date of tumor progression or the date of the last tumor assessment before subsequent cancer therapy.

Secondary end points included investigator-assessed progression-free survival (PFS), defined as the time from first dosing to the first documented tumor progression, as determined by investigators, or death because of any cause; duration of response (DOR), defined as the time from first confirmed tumor response (CR or PR) to tumor progression or death because of any cause, whichever occurs first; and overall survival (OS), defined as the time from first dosing to the date of death. Exploratory end points included safety and tolerability, as assessed by the frequency and severity of adverse events per the Common Terminology Criteria for Adverse Events version 4.0. Tumor assessments by computed tomography or magnetic resonance imaging were conducted at baseline, every 8 weeks during the first year, and every 12 weeks thereafter, until disease progression or treatment discontinuation. Survival was monitored at the first follow-up assessment 35 days after the final dose, 80 days after the first follow-up assessment, and every 3 months thereafter. Safety was monitored throughout the study and until 100 days after the final dose.

### Study Oversight

The study protocol was approved by an institutional review board or independent ethics committee at each site before study activation. The study was conducted in accordance with Good Clinical Practice guidelines, as defined by the International Conference on Harmonisation, and in accordance with the ethical principles of the European Union Directive and US Code of Federal Regulations. All patients provided written informed consent in accordance with the Declaration of Helsinki.

### Statistical Analysis

The planned sample size was 23 patients with MCC for the NIVO monotherapy cohort and 40 for the combination therapy cohort. At the time of planning this study, ORR data with PD-(L)1 blockade in patients with metastatic MCC were not yet available from other ongoing trials. In the monotherapy cohort, an ORR of > 10% was considered of clinical interest. If the true ORR is 20%, the probability of detecting three or more responses among 23 patients would be 86.7%; if the true ORR is 30%, the probability would be 98.4%. In the combination therapy cohort, an ORR of > 10% was considered of clinical interest. Assuming that the true ORR is 25%, 40 patients would provide approximately 79.8% power to reject the null hypothesis that the true ORR is 10%, considering a two-sided alpha of 5%.

## RESULTS

### Patient Characteristics and Treatments Received

Patients were enrolled at 26 sites in 10 countries (Belgium, France, Germany, Japan, the Netherlands, Republic of South Korea, Spain, Taiwan, United Kingdom, and United States), with five sites in the Netherlands, Spain, and United States enrolling in both cohorts. Enrollment of the NIVO monotherapy and NIVO + IPI cohorts was nonoverlapping; the NIVO monotherapy cohort was enrolled between October 15, 2015, and January 25, 2016, and the NIVO + IPI cohort was enrolled between July 19, 2016, and October 15, 2018. Because of this sequential enrollment, follow-up duration was longer for the monotherapy cohort than for the combination cohort. The first database lock (December 13, 2021) was the primary analysis database lock. The second database lock (November 28, 2022) was the study closeout database lock, whereupon the main efficacy analyses were updated, but efficacy subgroup analyses, including by previous lines of therapy, were not updated.

As of November 28, 2022, 68 patients had received either NIVO monotherapy (n = 25) or NIVO + IPI combination therapy (n = 43). The majority of patients had stage IV MCC at the time of study enrollment although a small number of patients had unresectable stage II or III disease, both in the NIVO cohort (n = 5, 20%) and in the NIVO + IPI cohort (n = 3, 7%). Most patients in both cohorts had previously received surgery and focal radiotherapy. In the NIVO cohort, the median age was 66 years (range, 27–88), 10 patients (40%) had an ECOG PS of 1, and 15 (60%) were treatment-naïve (Table 1). In the NIVO + IPI cohort, the median age was 70 years (range, 48–85), 27 patients (63%) had an ECOG PS of 1, and 33 (77%) were treatment-naïve (Table 1). The NIVO monotherapy cohort had a numerically lower proportion of patients with adverse prognostic factors than the combination treatment cohort, including age ≥65 years (60% v 74%, respectively), an ECOG PS of 1 (40% v 63%), stage IV disease (80% v 93%), virus-negative MCC (28% v 42%), and tumor burden at baseline (median, 55.5 v 72 mm; Table 1). However, the monotherapy cohort had a higher proportion of patients who had previously received systemic therapy for MCC than the combination cohort (40% v 23%). Among those who had received previous systemic therapy, most had received chemotherapy (Table 1).

Treatment duration with NIVO monotherapy exceeded that with NIVO + IPI. In the NIVO monotherapy cohort, the median treatment duration was 15.8 months (range, 0.03–62.2); in the combination cohort, the median treatment duration was 7.9 months (range, 0.03–33.15) for NIVO and 6 months (range, 0.03–30.85) for IPI. The median number of NIVO doses was 35 (range, 1–128) in the monotherapy cohort. By contrast, for the combination therapy cohort, the median number of doses for NIVO and IPI was 16 (range, 1–67) and 5 (range, 1–22), respectively. The median follow-up period was 62.5 months (range, 1.2–70.2) for monotherapy and 24.4 months (range, 0.8–57.3) for combination therapy.

The most common reasons for treatment discontinuation in both cohorts were disease progression (NIVO, 28%; NIVO + IPI, 33%) and unacceptable toxicity (NIVO, 20%; NIVO + IPI, 26%).

### Efficacy

ORRs were similar in both cohorts: 60% (95% CI, 38.7 to 78.9) for NIVO and 58% (95% CI, 42.1 to 73) for NIVO + IPI (Table 2). The median DOR (95% CI) was 60.6 months (16.7 to not applicable [NA]) with NIVO and 25.9 months (10.4 to NA) with NIVO + IPI. The median PFS (95% CI) and OS (95% CI), respectively, were 21.3 months (9.2 to 62.5) and 80.7 months (23.3 to NA) with NIVO and 8.4 months (3.7 to 24.3) and 29.8 months (8.5 to 48.3) with NIVO + IPI (Table 2).

**FIG 1.**
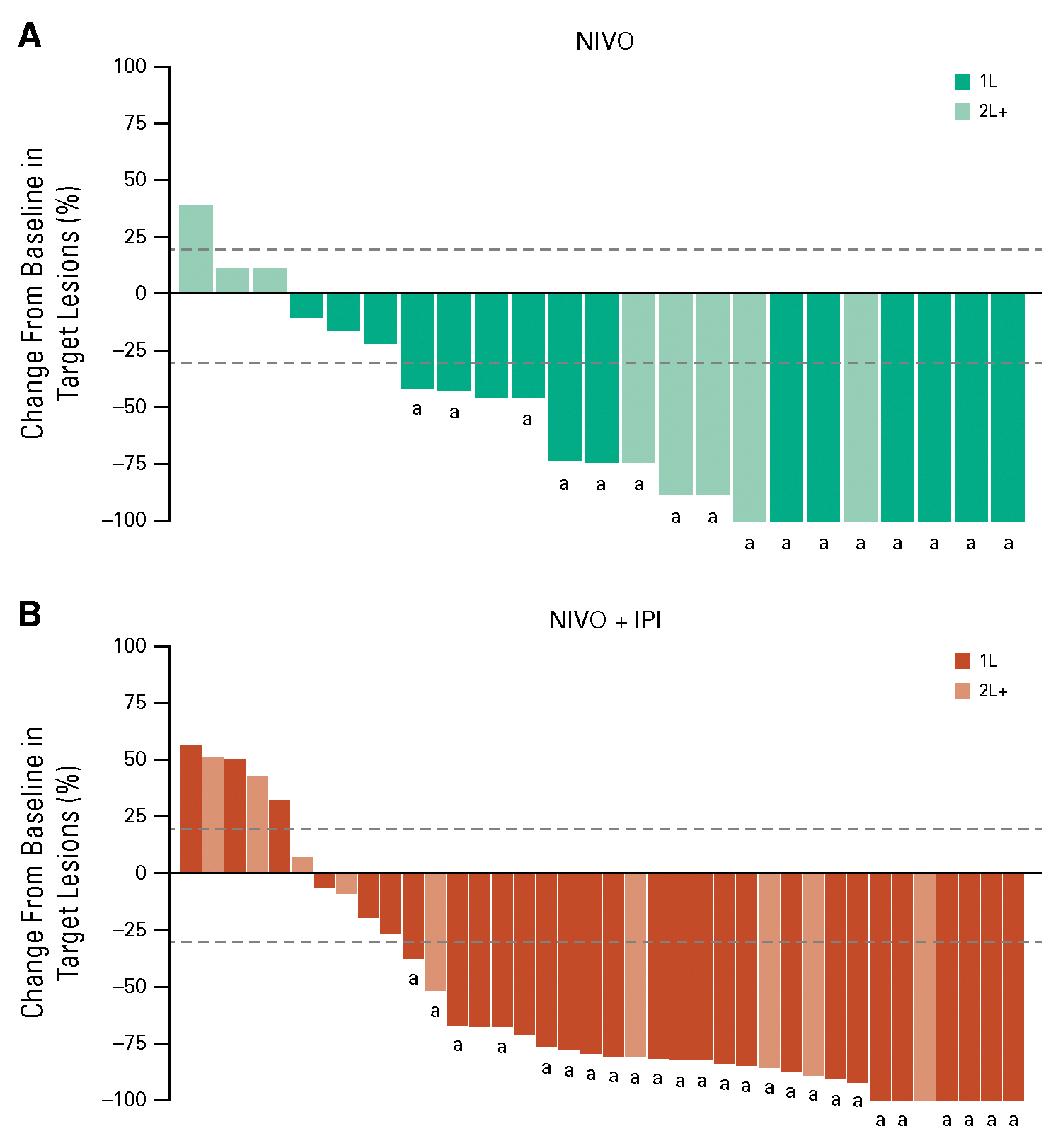
Change from baseline in target lesions in evaluable patients by previous line of therapy in the (A) NIVO and (B) NIVO + IPI treatment cohorts. Database lock: December 13, 2021. Horizontal dashed reference lines indicate 30% reduction and 20% increase consistent with a partial response and progressive disease, respectively, per RECIST version 1.1 criteria. Best overall response was unable to be determined in two patients in the NIVO 2L+ cohort, three patients in the NIVO + IPI 1L cohort, and one patient in the NIVO + IPI 2L+ cohort. Change from baseline in target lesion could not be determined in one patient with documented progressive disease in the NIVO + IPI 1L cohort. ^a^Patients who had a confirmed response (complete response or partial response). 1L, first line; 2L+, second or later line; IPI, ipilimumab; NIVO, nivolumab.

**FIG 2.**
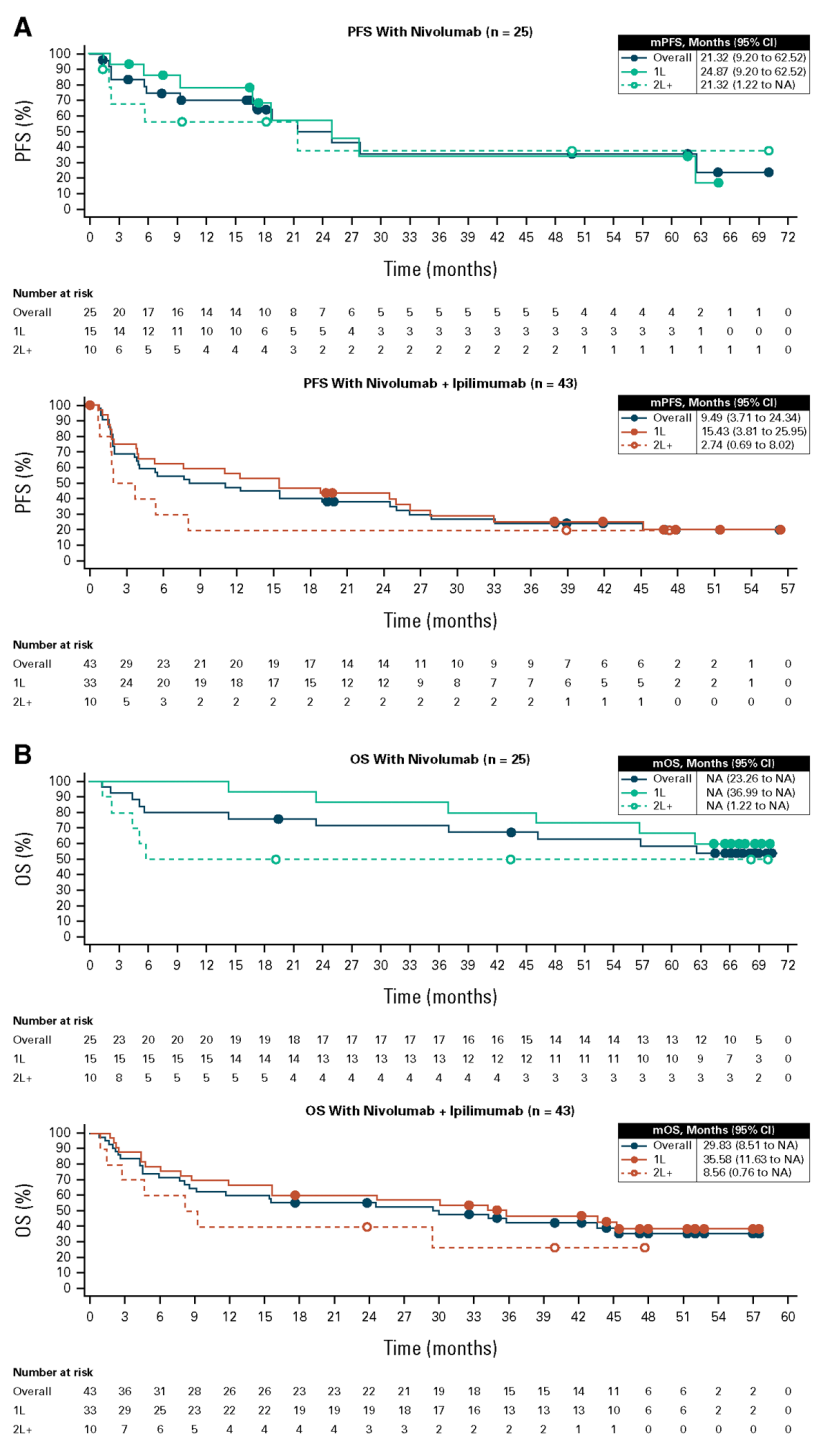
Kaplan-Meier curves of (A) PFS and (B) OS by treatment cohort and previous line of therapy. Database lock: December 13, 2021. Median follow-up was 62.5 months (range, 1.2–70.2) in the nivolumab cohort and 24.4 months (range, 0.8–57.3) in the nivolumab + ipilimumab cohort. 1L, first line; 2L+, second or later line; mOS, median OS; mPFS, median PFS; NA, not applicable; OS, overall survival; PFS, progression-free survival.

In both cohorts, ORRs appeared to be higher in treatment-naïve patients than in pretreated patients (Table 3, Figs 1A and 1B). Although patient numbers in the subgroups are small, median PFS in the NIVO cohort was similar among patients treated in the 1L and second or later line (2L+) settings; however, median PFS in the NIVO + IPI cohort appeared to be longer in patients treated in the 1L setting than in the 2L+ setting (Table 3, Fig 2A). Median OS also appeared to be longer in patients treated in the 1L setting than in the 2L+ setting, in both treatment cohorts (Table 3, Fig 2B).

### Safety

The incidence of grade 3/4 treatment-related adverse events (TRAEs) was numerically higher in the combination cohort (47%) than in the NIVO monotherapy cohort (28%) (Table 4). The incidence of treatment-related serious adverse events was also numerically higher with combination therapy (30%) than with monotherapy (8%). Nine of 25 patients (36%) in the NIVO cohort and 21 of 42 patients (50%) in the NIVO + IPI cohort required concomitant immunosuppressant use because of an immune-related adverse event. There was one treatment-related death in each cohort: pneumonitis in the NIVO cohort and GI motility disorder because of enteric neuropathy in the NIVO + IPI cohort, the latter of which has been reported elsewhere.^18^

Among patients who discontinued treatment for any reason other than disease progression, one of 18 patients (5.5%) in the NIVO cohort and two of 28 patients (7.1%) in the NIVO + IPI cohort subsequently experienced disease progression. One patient (4%) in the NIVO cohort and six patients (14.28%) in the NIVO + IPI cohort discontinued treatment because of toxicity and required concomitant immunosuppressant use; the DOR among these patients was 5.5 months for the patient in the NIVO cohort and 3.9, 17, 20.8, 21, 34.9, and 39.5 months for the six patients in the NIVO + IPI cohort.

## DISCUSSION

In this nonrandomized, multicohort, CheckMate 358 study, both NIVO monotherapy and the combination of NIVO + IPI were associated with frequent and durable responses in patients with ICI-naïve recurrent/metastatic MCC (ORR 60% and 58%, respectively, in the overall population and 73% and 64%, respectively, in treatment-naïve patients). However, the addition of IPI to NIVO did not appear to improve efficacy, despite evidence of increased immune activation from the combination therapy as reflected in increased toxicity in the combination cohort. To our knowledge, the CheckMate 358 combination therapy cohort of 43 patients, including 33 (77%) who were treatment-naïve, represents the largest prospective investigation of NIVO + IPI in patients with advanced MCC. These results contrast with the recently published study by Kim et al^17^ that reported an ORR of 100% among 24 ICI-naïve patients with advanced MCC who received the same regimen of NIVO + IPI as in our study.

This apparent lack of improvement in efficacy with the addition of IPI to NIVO in an immunogenic cancer like MCC is somewhat surprising although increasing evidence suggests that clinical benefit from adding anti–CTLA-4 to anti–PD-1 therapy may be specific to certain cancer types and treatment settings. For example, combination treatment with NIVO + IPI has shown improved efficacy compared with either agent alone in previously untreated advanced melanoma (CheckMate 067)^19^; however, in the adjuvant setting for resected stage IIIB-D or stage IV melanoma (CheckMate 915), adding IPI to NIVO therapy did not improve recurrence-free survival versus NIVO monotherapy.^20^ In a meta-analysis of 1,727 patients with eight different advanced cancer types other than melanoma who received NIVO monotherapy or NIVO + IPI, combination therapy was associated with a marginal improvement in PFS but no improvement in OS and resulted in significantly more grade 3/4 TRAEs and treatment-related discontinuations.^21^

In the current study, several adverse prognostic features (eg, age ≥65 years, an ECOG PS of 1, stage IV disease, greater tumor burden, virus-negative MCC) were represented in a relatively higher proportion of patients receiving NIVO + IPI than NIVO monotherapy. As these two patient cohorts were enrolled sequentially, it is plausible that the study investigators might have felt more comfortable enrolling patients with adverse prognostic factors in the combination therapy cohort than they did in the earlier monotherapy cohort because there was already growing evidence for the efficacy of NIVO and other anti-PD-(L)1 drugs in advanced MCC at that time. Interestingly, the distribution of most of the reported prognostic factors in the NIVO + IPI cohort in our study is quite similar to that of the ICI-naïve cohort in the other reported prospective MCC study by Kim et al.^17^

The higher incidence of grade 3/4 TRAEs, treatment-related serious adverse events, and treatment discontinuations observed in the NIVO + IPI cohort than in the NIVO cohort might have limited the duration of treatment and compromised the efficacy outcomes in our study. However, enhanced immune activation with ICI, as reflected by increased toxicity, is generally associated with improved clinical outcomes in other reports, as is evident from experience with ICI in patients with metastatic melanoma.^22–24^ This was not the case in the current study.

Our results also raise questions regarding the optimal dosing regimens of IPI in combination with NIVO. In CheckMate 915, adjuvant treatment with NIVO 240 mg once every 2 weeks plus IPI 1 mg/kg once every 6 weeks did not demonstrate an efficacy advantage over NIVO monotherapy in patients with resected stage IIIB-D or stage IV melanoma.^20^ Conversely, 1L treatment with NIVO 1 mg/kg plus IPI 3 mg/kg once every 3 weeks for a total of four doses demonstrated a clinically relevant efficacy advantage over NIVO monotherapy in patients with unresectable stage III or stage IV melanoma.^19^ Consequently, it could be argued that the dose of IPI used in this study (1 mg/kg once every 6 weeks) should have been higher (eg, 3 mg/kg) or administered more frequently (eg, once every 3 weeks for a total of four doses), similar to regimens used in advanced melanoma.^25^ However, as the median age of patients with advanced MCC generally exceeds that for advanced melanoma, there were significant concerns about the tolerance of serious toxicities in this generally elderly patient population. Of note, the dosing regimen used in our study was identical to that in the phase II study by Kim et al,^17^ which reported an ORR of 100% in ICI-naïve patients, albeit in a smaller cohort of patients (n = 24) enrolled at only two centers, which could have introduced inadvertent selection bias. While the discrepant results between these two studies do not have an obvious explanation, they demonstrate the need for a prospective randomized trial to definitively compare NIVO + IPI versus NIVO monotherapy in patients with advanced MCC in the 1L or later-line treatment settings. Such an investigation may be worthwhile for improving MCC outcomes as increasing retrospective and prospective data suggest that IPI is an active agent in MCC, including in the ICI-refractory treatment setting. It may also be worthwhile to evaluate NIVO in combination with inhibitors of other immune checkpoints, such as anti–lymphocyte-activation gene 3 (LAG-3) and anti-T-cell immunoglobulin and mucin domain 3 (TIM-3), which may be contributing to immune evasion in PD-L1–resistant MCC.^26,27^ Our current study, as well as the previous study by Kim et al,^17^ provides essential data to guide the assumptions for designing a future randomized trial, which will require extensive collaboration among academic investigators and the MCC patient community.

Our study has several limitations. First, as this was a non-randomized study, direct quantitative comparisons between NIVO monotherapy and NIVO + IPI cannot be made. Second, the sample size in each of the cohorts was relatively small compared with studies of other tumor types although this was expected given the rarity of MCC. Third, the non-randomized study design and sequential enrollment of the NIVO and NIVO + IPI cohorts led to apparent imbalances in key prognostic features between the two treatment cohorts and resulted in a longer duration of follow-up in the NIVO monotherapy cohort. Nevertheless, the high ORR, associated with long DOR, PFS, and OS, in the NIVO monotherapy cohort of our study is consistent with the observed efficacy of other anti–PD-(L)1 agents that are FDA-approved as monotherapies for advanced MCC and highlights the overall improvement in outlook for patients with advanced MCC after the advent of PD-1 pathway blockade.

In summary, our nonrandomized CheckMate 358 study shows that both NIVO monotherapy and the combination of NIVO + IPI are associated with frequent and durable responses in patients with ICI-naïve recurrent/metastatic MCC. However, the results do not suggest additional efficacy from combining IPI with NIVO in treating advanced MCC. Given the conflicting results from the study by Kim et al, a prospective randomized trial of NIVO + IPI versus anti-PD-(L)1 monotherapy in patients with MCC is warranted to investigate this definitively.

## Figures and Tables

**TABLE 1.** Patient Demographics and Baseline Characteristics of All Treated Patients

Patient Characteristic	NIVO (n = 25)	NIVO + IPI (n = 43)
Age, years, median (range, min-max)	66 (27–88)	70 (48–85)
≥18 and <65, No. (%)	10 (40)	11 (26)
≥65 and <85, No. (%)	14 (56)	30 (70)
≥85, No. (%)	1 (4)	2 (5)
Race, No. (%)		
White	23 (92)	36 (84)
Asian	1 (4)	5 (12)
Other	1 (4)	2 (5)
Sex, No. (%)		
Female	8 (32)	10 (23)
Male	17 (68)	33 (77)
ECOG PS, No. (%)		
0	15 (60)	16 (37)
1	10 (40)	27 (63)
Region, No. (%)		
United States/Canada	9 (36)	19 (44)
Europe	16 (64)	20 (47)
Rest of the world	0	4 (9)
Stage AJCC 7th edition, No. (%)		
Stage II^a^	0	2 (5)
Stage III^a^	5 (20)	1 (2)
Stage IV	20 (80)	40 (93)
Previous systemic therapy regimens in metastatic setting, No. (%)^b^		
0	15 (60)	33 (77)
1	7 (28)	8 (19)
2	3 (12)	2 (5)
MCPyV T-Ag serology (AMERK) test, No. (%)^c^		
Positive	11 (44)	14 (33)
Negative	7 (28)	18 (42)
Borderline	1 (4)	1 (2)
Not tested	6 (24)	10 (23)
Sum of diameters of target lesions, mm, median (range)	55.5 (11.1–198)	72 (21–266)

NOTE. Database lock: December 13, 2021.

Abbreviations: AJCC, American Joint Committee on Cancer; AMERK, Anti Merkel Cell Panel; ECOG PS, Eastern Cooperative Oncology Group performance status; IPI, ipilimumab; MCC, Merkel cell carcinoma; MCPyV, Merkel cell polyomavirus; NIVO, nivolumab.

a All patients with stage II and III MCC had unresectable disease.

b Previous systemic therapy for metastatic disease included carboplatin, cisplatin, cyclophosphamide, doxorubicin, etoposide, fluorouracil, lanreotide, and vincristine in the NIVO cohort and carboplatin, cisplatin, cyclophosphamide, doxorubicin, etoposide, everolimus, investigational antineoplastic ABBV-075, paclitaxel, pazopanib, and vincristine in the NIVO 1 IPI cohort.

c Viral serotyping of blood samples was analyzed to assess MCPyV association of patients’ MCC tumors. Small T antibody standard titer units reference ranges: <75 is negative, 75–150 is borderline, and >150 is positive.^28^

**TABLE 2.** Efficacy Results by Treatment Cohort

Efficacy End Point	NIVO (n = 25)	NIVO + IPI (n = 43)
ORR,^a^ % (95% CI) No.	60 (38.7 to 78.9) 15	58 (42.1 to 73) 25
CR, No. (%)	8 (32)	8 (19)
PR, No. (%)	7 (28)	17 (40)
SD, No. (%)	5 (20)	4 (9)
PD, No. (%)	3 (12)	10 (23)
NE, No. (%)^b^	2 (8)	4 (9)
PFS,^a^ months, median (95% CI)	21.3 (9.2 to 62.5)	8.4 (3.7 to 24.3)
DOR,^a^ months, median (95% CI)	60.6 (16.7 to NA)	25.9 (10.4 to NA)
OS, months, median (95% CI)	80.7 (23.3 to NA)	29.8 (8.5 to 48.3)
Patients with DOR of at least		
12 months, No. (%)	12 (80)	17 (68)
18 months, No. (%)	8 (53)	15 (60)
24 months, No. (%)	6 (40)	13 (52)

NOTE. Database lock: November 28, 2022. One patient in the NIVO cohort who was originally classified as a responder in the December 13, 2021 database lock (see Table 3) was subsequently reclassified as a nonresponder in this final database lock.

Abbreviations: CR, complete response; DOR, duration of response; IPI, ipilimumab; NA, not applicable; NE, not evaluable; NIVO, nivolumab; ORR, objective response rate; OS, overall survival; PD, progressive disease; PFS, progression-free survival; PR, partial response; SD, stable disease.

a ORR, DOR, and PFS were investigator-assessed.

b A postbaseline scan was unavailable for these patients.

**TABLE 3.** Efficacy Results by Treatment Cohort and Previous Line of Therapy

	NIVO		NIVO + IPI	
Efficacy End Point	1L (n = 15)	2L+ (n = 10)	1L (n = 33)	2L+ (n = 10)

ORR,^a^ % (95% CI) No.	73 (45 to 92) 11	50 (19 to 81) 5	64 (45 to 80) 21	40 (12 to 74) 4

CR, No. (%)	6 (40)	2 (20)	7 (21)	1 (10)

PR, No. (%)	5 (33)	3 (30)	14 (42)	3 (30)

SD, No. (%)	3 (20)	1 (10)	3 (9)	1 (10)

PD, No. (%)	1 (7)	2 (20)	6 (18)	4 (40)

NE, No. (%)^b^	0	2 (20)	3 (9)	1 (10)

PFS,^a^ months, median (95% CI)	24.9 (9.2 to 62.5)	21.3 (1.2 to NA)	15.4 (3.8 to 26)	2.7 (0.7 to 8)

DOR,^a^ months, median (95% CI)	43.2 (16.7 to NA)	NA (19.3 to NA)	25 (12.2 to NA)	NA (3.8 to NA)

OS, months, median (95% CI)	NA (37 to NA)	NA (1.2 to NA)	35.6 (11.6 to NA)	8.6 (0.8 to NA)

Patients with DOR of at least				

12 months, No. (%)	9 (82)	4 (80)	16 (76)	2 (50)

18 months, No. (%)	5 (45)	3 (60)	12 (57)	2 (50)

24 months, No. (%)	4 (36)	2 (40)	10 (48)	2 (50)

NOTE. Database lock: December 13, 2021. One responder in the NIVO cohort was subsequently reclassified as a nonresponder at the final database lock (see Table 2).

Abbreviations: 1L, first line; 2L1, second or later line; CR, complete response; DOR, duration of response; IPI, ipilimumab; NA, not applicable; NE, not evaluable; NIVO, nivolumab; ORR, objective response rate; OS, overall survival; PD, progressive disease; PFS, progression-free survival; PR, partial response; SD, stable disease.

a ORR, DOR, and PFS were investigator-assessed.

b A postbaseline scan was unavailable for these patients.

**TABLE 4.** Summary of TRAEs

	NIVO (n = 25), No. (%)		NIVO + IPI (n = 43), No. (%)	
Adverse Event	Any Grade	Grade 3/4	Any Grade	Grade 3/4

Any TRAE	21 (84)	7 (28)	36 (84)	20 (47)

Any TRAE occurring in ≥15% of patients^a^				

Fatigue	9 (36)	0	21 (49)	2 (5)

Increased lipase	7 (28)	5 (20)	9 (21)	6 (14)

Rash	5 (20)	0	14 (33)	2 (5)

Pruritus	5 (20)	0	17 (40)	0

Increased amylase	5 (20)	0	6 (14)	3 (7)

Pneumonitis	4 (16)	1 (4)	4 (9)	0

Asthenia	4 (16)	0	1 (2)	0

Diarrhea	3 (12)	0	11 (26)	0

Arthralgia	3 (12)	0	7 (16)	1 (2)

Hypothyroidism	2 (8)	0	7 (16)	0

SAEs	12 (48)		28 (65)	

TRSAEs	2 (8)		13 (30)	

Discontinuation because of study drug toxicity^b^	5 (20)		11 (26)	

Study drug-related deaths	1 (4)^c^		1 (2)^d^	

NOTE. Database lock: November 28, 2022.

Abbreviations: IPI, ipilimumab; NIVO, nivolumab; SAE, serious adverse event; TRAE, treatment-related adverse event; TRSAE, treatment-related SAE.

a Data shown are the incidence of each TRAE at the indicated grade in the indicated treatment cohort.

b TRAEs leading to discontinuation in the NIVO cohort were pneumonitis (n = 2), diarrhea (n = 1), pancreatic failure (n = 1), and colitis (n = 1). TRAEs leading to discontinuation in the NIVO 1 IPI cohort were decreased weight, pruritus, immune-mediated hepatitis, myositis, dizziness, GI motility disorder, liver function test increased, pancreatitis, ALT increased, abdominal pain, and adrenal insufficiency (all n = 1).

c Pneumonitis.

d GI motility disorder because of enteric neuropathy.^18^

## Data Availability

Data may be obtained from a third party and are not publicly available. Bristol Myers Squibb will honor legitimate requests for clinical trial data from qualified researchers with a clearly defined scientific objective. Data sharing requests will be considered for phase II-IV interventional clinical trials that were completed on or after January 1, 2008. In addition, primary results must have been published in peer-reviewed journals and the medicines or indications must have approved in the US, EU, and other designated markets. Sharing is also subject to protection of patient privacy and respect for the patient’s informed consent. Data considered for sharing may include nonidentifiable patient-level and study-level clinical trial data, full clinical study reports, and protocols. Requests to access clinical trial data may be submitted using the enquiry form at https://vivli.org/ourmember/bristol-myers-squibb/.

## CONTEXT

### Key Objective

To investigate the efficacy and tolerability of nivolumab (NIVO) with or without ipilimumab (IPI) in patients with Merkel cell carcinoma.

### Knowledge Generated

Treatment with NIVO with or without IPI led to frequent and durable responses: the objective response rate and median duration of response (DOR), respectively, were 60% and 60.6 months with NIVO monotherapy and 58% and 25.9 months with NIVO + IPI. NIVO + IPI was associated with a high incidence of grade 3/4 adverse events, which might have limited treatment duration and compromised efficacy outcomes.

### Relevance *(G. McArthur)*

Data provided suggests combined IPI and NIVO does not provide higher response rates nor longer DOR than single agent NIVO, but does have higher grade 3/4 toxicities. Based on these data combination immunotherapy cannot be routinely recommended.*

*Relevance section written by JCO Associate Editor Grant McArthur, MBBS, PhD.
